# Geographical epidemiology of antibacterials in the preschool age

**DOI:** 10.1186/1476-072X-11-52

**Published:** 2012-12-14

**Authors:** Massimo Cartabia, Rita Campi, Antonio Clavenna, Angela Bortolotti, Ida Fortino, Luca Merlino, Maurizio Bonati

**Affiliations:** 1 Laboratory for Mother and Child Health, Department of Public Health, “Mario Negri” Pharmacological Research Institute, Via Giuseppe La Masa 19, 20156, Milan, MI, Italy; 2 Lombardy Region Health Ministry, Regione Lombardia, Piazza Città di Lombardia 1, 20124, Milan, MI, Italy

**Keywords:** Geographic information systems, Spatial autocorrelation, Spatial clusters, Drugs prescriptions, Pediatrics

## Abstract

**Abstract:**

**Background**

Thematic maps allow a more rapid and immediate reading of the geographical differences in the distribution of data referred to a specific territory. The aim of this study was to show, for the first time, the application of some statistical and cartographic tools in the analysis of drug utilization in the pediatric population of an Italian region, and to assess the intra-regional difference in the antibiotic prescriptions.

**Methods**

To assess the type of geographic distribution of the prescriptions, the analyses were based on the standardized prevalence rate (z-score) calculated at the local health unit, health district, and municipality levels. Pearson’s coefficient of correlation was used to evaluate the correlation with hospitalization and the Moran’s I index was used to evaluate the existence of spatial autocorrelation. With the use of Getis-Ord’s G statistic, clusters of areas with high and low levels of prevalence were identified and mapped. The probability of receiving at least one prescription of antibacterials during the year for all the children included in the study was evaluated with a logistic regression model.

**Results**

With the use of the maps it was possible to see that the prescriptions were not correlated with the health status of the population, but with the tendency of the pediatrician to prescribe drugs. This was also confirmed by the logistic regression model constructed to estimate the probability of receiving at least one prescription of antibacterials considering, as independent variables: age, sex, prevalence of hospitalizations in the district of residence, prescriptive attitude of the pediatrician, sex of the pediatrician, pediatrician’s age group, and duration of the pediatrician’s contract with the local health unit (LHU).

**Conclusions**

The priority actions to rationalize the use of antibacterials in the preschool age should concentrate on the active participation of the pediatricians in permanent education activities. Moreover, the competent authorities should increasing their efforts to limit unnecessary prescriptions and increase appropriateness of prescribing.

**Riassunto:**

**Introduzione**

Le mappe tematiche consentono una più rapida ed immediata lettura delle differenze geografiche nella distribuzione di dati riferiti ad un territorio specifico. Lo scopo dello studio è mostrare, per la prima volta, l’applicazione di alcuni strumenti statistici e cartografici, nell’analisi dell’uso dei farmaci nella popolazione pediatrica di una regione italiana e valutare le differenze intra-regionali.

**Metodi**

Per valutare il tipo di distribuzione geografica delle prescrizioni, sono stati calcolati i tassi di prevalenza standardizzati (punteggi-z) a livello di ASL, Distretti Sanitari e Comuni. Per valutare la correlazione con le ospedalizzazioni è stato usato il coefficiente di correlazione di Pearson; per valutare l’esistenza di autocorrelazione spaziale è stato usato l’indice I di Moran. Tramite l’uso della statistica G di Getis-Ord sono stati identificati cluster di aree ad alto e basso livello di prevalenza. Infine con un modello di regressione logistica è stata stimata la probabilità di ricevere almeno una prescrizione nel corso dell’anno per tutti i pazienti inclusi nello studio.

**Risultati**

Con l’uso delle mappe è possibile vedere che le prescrizioni non sono correlate con lo stato di salute della popolazione, ma sono correlate con l’attitudine prescrittiva del pediatra. Questo è confermato anche dal modello di regressione logistica costruito per stimare la probabilità di ricevere almeno una prescrizione considerando come variabili indipendenti l’età, il sesso, la prevalenza di ricoveri nel distretto di residenza, l’attitudine prescrittiva del pediatra, la classe di età del pediatra e la durata della convenzione del pediatra con l’Azienda Sanitaria Locale (ASL).

**Conclusioni**

Gli interventi primari per razionalizzare l’uso degli antibiotici in età prescolare dovrebbero concentrarsi sulla partecipazione attiva dei pediatri in attività di educazione permanente. Inoltre il contenimento delle prescrizioni di ASL e Distretti dovrebbe prevedere un ulteriore controllo da parte delle autorità competenti in merito all’appropriatezza prescrittiva.

## Background

Antibacterials are the drugs most frequently prescribed to children, especially preschoolers, and are often prescribed for diseases (e.g. colds, upper respiratory tract infections, bronchitis) that do not benefit from antibiotic therapy [1]. About 50% of antibacterial prescriptions for children are inappropriate [2-4]. Overuse of antibacterials can lead to increased adverse reactions and bacterial resistance [5]. The increase in bacterial resistance is a global public health problem, both in terms of social costs and consequences on health [6-8]. There are important quantitative and qualitative differences in the prescription of antibiotics to the general and pediatric populations, both nationally and internationally [9,10]. Italian children receive antibiotic prescriptions more often than Dutch or English children [9]. Cephalosporins are widely prescribed in Italy, while they represent less than one percent of the pediatric prescriptions in Denmark and the Netherlands. Significant inter- and intra-regional differences in the prescription profiles in Italy have also been found. A study conducted in 8 Italian regions documented that the prevalence of antibiotic prescribing varied, depending on the region, from 43% to 62% [10]. The difference is even greater at the Local Health Unit (LHU) level, with a prevalence ranging from 36% to 69% [10]. The prevalence of use of antibacterials is lower in the regions of Northern Italy and greater in the South. In contexts characterized by a greater prescription of drugs, higher prescriptions of medications considered as second choice were also observed [10]. The Geographic Information Systems (GIS), which are software designed to create maps and make spatial analyses, are also being widely employed in the health sector, in both research and programming. In all those who work in public health, there has been an increased interest in GIS and its characteristics, in the knowledge of the variability between different geographical areas according to environmental, socioeconomic, and demographic factors, in order to permit the programming to be more focused at the local level and to identify “problem areas” that require priority actions [11]. Some of the main analyses in which these systems can be applied are: population and health needs, environmental risk factors, and the localization of health services and facilities in the territory. Geographical differences may be related to several factors, among which are demographic factors and socio-cultural attitudes of prescribing physicians. The knowledge of the determinants of antibacterial prescribing is therefore essential in the planning of training and educational interventions for doctors and citizens. In this regard, the use of thematic mapping and spatial analysis [12] can be a useful aid for the representation of territorial differences [10]. A study was therefore conducted, for the first time in an Italian region, to assess the intra-regional differences in prescriptions of antibacterials to preschool children at different territorial levels (LHU, health district, and municipality) considering also the individual prescriber.

## Methods

### Organization of the Italian national health service

The Italian National Health Service is divided into 145 local health units (LHUs) which are, in turn, divided into health districts, which include one or more municipalities. In the Lombardy Region there are 15 LHUs, 98 health districts, and 1,546 municipalities. Each district includes an average of about 16 municipalities and about 6,500 preschool age children (between 0 and 6 years). The municipality of Milan is an exception, since it is a very large city, and is divided into five health districts. Every Italian resident is registered with a family (pediatric or general) practitioner. Children are assigned to a pediatrician until they are 6 years old; afterwards, the parents can choose to register a child with a general practitioner. A national formulary is available, in which drugs are categorized into two classes: class A includes essential drugs that patients do not have to pay for and class C contains drugs not covered by the National Health Service (NHS). Nearly all antibacterials are free of charge.

### Source data and software

Data are stored in administrative databases [13] that contain all the prescription, hospitalization, and administrative information about individuals living in the Lombardy Region up to the year 2008, the last year for which data is currently available for these analyses.

To localize the pediatricians, the websites of the 15 LHUs of the Lombardy Region, which contain office addresses, were consulted. These addresses were then converted into UTM coordinates. The statistical analyses were performed with SAS 9.1.3 [14] and the cartographic representations and spatial analyses with ArcMap 10 [15].

### Population selection

Specific selection criteria were defined for the inclusion of patients in the study in order to avoid the presence of pediatricians or territorial areas with few patients and high prevalence rates of prescriptions:

1) patients aged between 0 and 6 years on 31 December 2008.

2) family pediatricians in charge of a minimum of 100 patients aged between 0 and 6 years.

3) municipalities with at least 6 patients aged 0–6 years assisted by a pediatrician in charge of at least 100 patients aged 0–6 years. Six was the minimum number of children needed to identify at least one antibacterial user, given a prevalence of antibacterial prescriptions in the population of 50%.

With these criteria, the selected population represented 95% of children living in the Lombardy Region aged between 0 and 6 years on 31 December 2008. The prevalence of patients with at least one antibacterial prescription (identified by the code J01 of the Anatomical Therapeutic Chemical Classification System) in the year 2008 was calculated on the total number of patients resident in the Lombardy Region in 2008. The rate was then stratified for the 15 LHUs, for the 98 health districts, and, finally, for the 1,546 municipalities of the Lombardy Region.

The representation of the rates was made with choropleth maps, based on the standard deviations of the rates (z-scores) to best evaluate the territorial differences. To verify the model of geographical distribution, the Moran's I index of spatial autocorrelation [16] was used, which assesses whether the areas with similar values of prescription prevalence are clustered, dispersed, or distributed randomly in the territory. The statistically significant concentrations of areas with high and low prevalence of prescriptions were found by calculating, for each territorial area, the Getis-Ord’s G statistic [16] and graphically representing statistically significant clusters at the α = 0.05 level.

### Selection of hospitalizations

To see whether the prescription rate is associated with the health status of the population, the correlation with the prevalence rate of patients with at least one hospitalization during 2008 was calculated. All hospitalizations were considered except those:

of healthy infants (identified by ICD9 code V3X);

that occurred in the first week of life of the patient;

for injury or poisoning (ICD9 codes identified by the range E800-E999).

The correlation with the prescription rate was evaluated by calculating the Pearson correlation coefficient, at both the health district and municipality levels.

### Analysis of prescribers

For each pediatrician with at least 100 patients aged 0–6 in 2008 and correctly localized in the database, 4 indicators were calculated in order to establish whether the pediatricians that prescribe many antibacterials are the same as those who prescribe many drugs in general. The 4 indicators were:

A) The percentage of patients with at least one drug prescription on the total number of patients registered with the pediatrician.

B) The percentage of patients with at least one prescription of antibacterials on the total number of patients registered with the pediatrician.

C) The mean number of prescriptions per patient, considering all types of drugs, over the total number of patients registered with the pediatrician.

D) The mean number of prescriptions of antibacterials, over the number of patients registered with the pediatrician who received at least one antibacterial prescription.

The Pearson’s Chi-Square Coefficient of Correlation between A and B was then evaluated, followed by that between C and D. The second indicator (B) was considered as an index of tendency to prescribe antibacterials. This indicator was standardized in order to create 3 categories of pediatricians: the “Low prescribers” with z <−1, the “Mean prescribers” with −1 ≤ z ≤ 1, and the “High prescribers” with z > 1. Finally, the map of the prevalence rates and the map with the positions of the High and Low prescribers were overlapped to make spatial comparisons.

### Logistic regression model

A logistic regression model was built to estimate the probability of receiving at least one prescription of antibiotics by using as independent variables: age, sex, prevalence of hospitalizations in the district of residence, prescriptive attitude of the pediatrician, sex of pediatrician, pediatrician age group, and duration of the pediatrician’s contract with the LHU. Depending on the model obtained, the probability of receiving at least one prescription of antibiotics during the year was then calculated for each patient.

### Definition of GIS (Geographical Information System)

GIS is an acronym for Geographical Information System, which is an application designed as a set of interacting parts, capable of producing information on the territory and then georeferenced. A GIS is made up of hardware and software used in the treatment of geographic information; it requires an organized set of rules and human and material resources that interact dynamically, allowing the various functions of collection, storage, management, recovery, conversion, data analysis and modeling, visualization, and communication of information that has specific geographical significance. An important characteristic of GIS is its ability to integrate data from multiple sources; the data may vary in size, type, and structure and can generally be summarized into two categories: spatial data (in vector and raster) and attributes. The spatial data, or graphical objects, correspond to polygons, lines, points, pixels, symbols and annotations. Objects are, for example: the geographical demarcation of a province and the topography of an area or a road network. The attributes, on the other hand, correspond to the characteristics of the geographical objects, for example: distribution of the population of an area, disease prevalence and users of a service. Geocoding, or geotagging, is the process by which the GIS combines a unique geographical reference to an object, uniquely positioning it on a map, either explicitly by the coordinates (x, y) or implicitly through, for example, the address or zip code. A distinctive, versatile aspect of GIS is the ability to overlay data layers or strata, each of which describes a category of information (for example: roads, location of hospitals and health centers, morbidity or mortality). This process allows the performance of different types of analysis and construction of digital maps. An essential element for analysis is the geographical scale that is the size of the area of interest. The geographic scope of analysis can vary from very large areas (nations, regions) to smaller areas (LHU, town, city), to levels of district, block, street, house number.

### The choropleth thematic maps

A choropleth map represents an indicator with a chromatic scale that is divided into classes and refers to polygonal units of observation. In this study the standardized distribution of the indicator is used to create the intervals of the scales of the indicators. The standardization is a statistical technique that transforms the original distribution into a distribution with a mean value of 0 and a standard deviation of 1. The standardized value of the single unit of observation is called “z-score” and is calculated with the formula: Z=(X-μ)/σ (where: X= value of the original variable, μ= mean of the values of the original variable, σ= standard deviation of the values of the original variable). Usually, if |Z| is higher than 1, the unit of observation is a significant deviation from the mean.

The p-value of the G statistic of the single area was used to map the spatial clusters. Since clusters refer to a confidence level, α=0.05 was used, so if Z(Gi) > 1.96 the area is part of a cluster of high values and if Z(Gi) < −1.96 the area is part of a cluster of low values.

## Results

Table 1 summarizes the target population data.

**Table 1:** Frequencies of the considered variables

**Territorial Level**	**Territorial Units N**	**Pediatricians Mean (range)**	**Residents Mean (range)**	**Patients Mean (range)**	**Prevalence (%) Mean (range)**	**Prescriptions Mean (range)**
Lombardy Region	1	1,151	626,894	347,932	55.5	880,287
LHU	15	76.7 (10 – 167)	41,792.9 (5,982 – 90,345)	23,195.5 (3,603 – 46,844)	55.6 (47.8 – 60.7)	58,685.8 (9,522 – 135,830)
Health Districts*	97	11.9 (1 – 36)	6,462.8 (735 – 20,529)	3,586.9 (463 – 9,516)	56 (36.7 – 66.6)	9,075.1 (1,036 – 22,118)
Municipalities*	1,497	0.8 (0 – 134)	418.8 (6 – 74,162)	232.6 (1 – 34,084)	55.3 (0 – 100)	588.4 (1 – 78,655)

*1 health district and 49 municipalities are excluded from the analyses.

### Analysis of the prescriptions at the LHU level

The prevalence rate of the prescription of antibacterials in the 15 LHUs of the Lombardy Region varies from a minimum of 47.8% to a maximum of 60.7%, its mean is 55.6%, and the standard deviation is 3.8. The Moran’s I Index was calculated to understand if there is a significant spatial autocorrelation, and its value was −0.02, corresponding to a z-score of 0.34 (p-value = 0.73); no spatial autocorrelation was therefore found.

### Analysis of the prescriptions at the health district level

**Figure 1:**
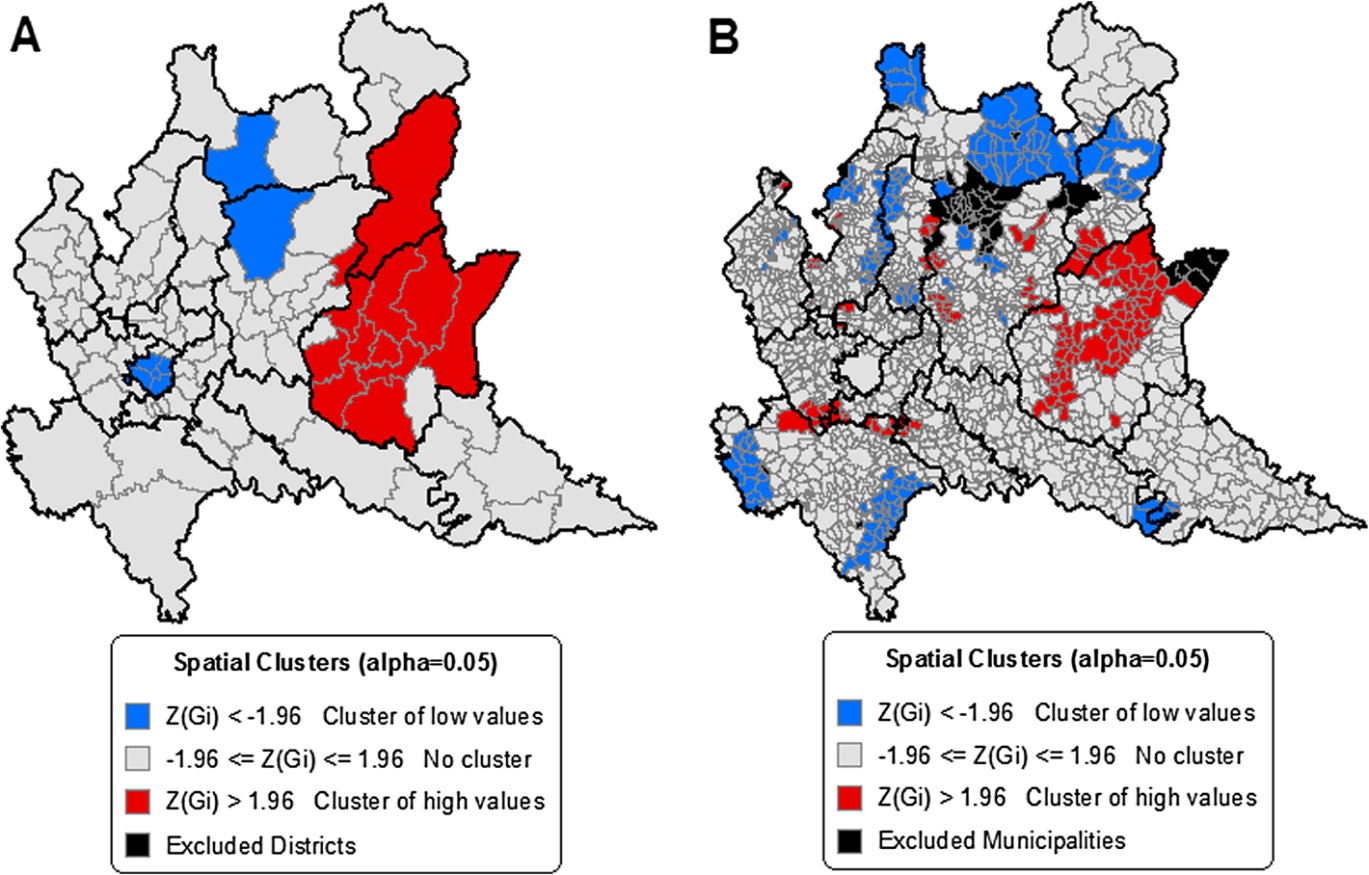
Territorial clusters of the prevalence rate of prescriptions at the health district and municipality level.

The prevalence rate of antibacterial prescriptions in the 97 health districts of the Lombardy Region included in the study varies from a minimum of 36.7% to a maximum of 66.6%, with a mean of 56.0% and a standard deviation of 5.4. The Moran’s I Index is equal to 0.27, corresponding to a z-score of 4.70 (p-value <0.0001), meaning that there is a significant spatial autocorrelation, and that it may be possible to find spatial clusters. The value of Moran’s I Index is not very high, but the z test confirms that it is significant and significant spatial clusters, in fact, have been found. Map 1A in Figure 1 shows two clusters of health districts with low values of prescription rates: one in the north of the region and the other that corresponds to the City of Milano. There is also a large cluster of health districts with high values of prescription rates represented by the Vallecamonica-Sebino LHU and almost the entire LHU of Brescia. The data obtained for this study can be inspected in more detail and the distribution of the prevalence rate of prescriptions at the municipal level can be evaluated to understand if the spatial autocorrelation remains or disappears.

### Analysis of the prescriptions at the municipality level

The prevalence rate of prescriptions of antibacterials for the 1,497 municipalities of the Lombardy Region included in the study varies between 0.0% and 100.0%, with a mean of 55.3% and a standard deviation of 9.9. The Moran’s I Index is 0.35, corresponding to a z-score of 22.61 (p-value <0.0001). This result means that the presence of spatial autocorrelation, already found at the health district level, also exists, and is even greater, at the municipal level. Different spatial clusters can be seen when analyzing map 1B in Figure 1 of the spatial clusters at the municipal level. The biggest cluster is located in the eastern part of the region and is formed by 40% of the municipalities of the Brescia LHU, plus other neighboring municipalities of Vallecamonica-Sebino and Bergamo LHUs, which have high prevalence rate values. Another big cluster of municipalities, but with low prevalence rate values, is located in the northern part of the region and is formed by 36% of the municipalities of the Sondrio LHU, plus other neighboring municipalities of the Vallecamonica-Sebino LHU. Other smaller clusters of municipalities with both high and low prevalence rate values are dispersed in the central and southern parts of the region.

Comparing the values of the Moran’s I Index at the municipal and health districts levels, it’s possible to see an increase in the spatial autocorrelation. Comparing the three geographical levels, the value of the Moran’s I Index increases from −0.02 for the 15 LHUs to 0.35 for the 1,497 municipalities: there are therefore elements clearly related to the territory that influence the geographic distribution of the prescriptions of antibacterials. With the data on the hospitalizations obtained for this study, it is also possible to verify whether there is a correlation with the health status of the population.

### Analysis of the hospitalizations

In order to evaluate the health status of the population, the prevalence rate of hospitalizations was calculated considering the hospitalizations of all the patients included in the study, based on the selection criteria presented in the “Methods” section. The rate, stratified by health district, had a mean of 8.0% and a standard deviation of 1.3. The raw rate was standardized and the districts were then divided into 3 classes, based on the standard deviation. The class of the districts with a low prevalence of hospitalizations included those with a prevalence rate lower than 6.7%. The class with an average prevalence of hospitalizations included those with a prevalence rate of between 6.7% and 9.3%. The districts with a prevalence rate of hospitalizations higher than 9.3% were included in the class of the districts with a high prevalence of hospitalization. Similarly, the prevalence rate of hospitalizations stratified by municipality was calculated, and had a mean of 7.8% and a standard deviation of 3.4. The municipalities with a low prevalence had a rate lower than 4.4%, those with an average prevalence had a rate of between 4.4% and 11.2%, and those with a high prevalence have a rate higher than 11.2%. The health districts and municipalities were classified with the same method based on the z-score of the prevalence rate of prescriptions of antibacterials to evaluate the correlation with the hospitalizations. The results are in Table 2.

**Table 2:** Levels of the standardized prevalence rates of hospitalizations and prescriptions of antibacterial

**Health Districts**^**#**^					**Municipalities**^**#**^				
Hospitalizations*	Prescriptions*			**Total**	Hospitalizations*	Prescriptions*			**Total**
	**Low**	**Mean**	**High**			**Low**	**Mean**	**High**	
**Low**	**4**	9	1	14	**Low**	**55**	107	13	175
**Mean**	8	**52**	9	69	**Mean**	119	**889**	130	1,138
**High**	3	8	**3**	14	**High**	26	115	**43**	184
**Total**	15	69	13	97	**Total**	200	1,111	186	1,497

* The 3 classification levels are based on the z-score of the 2 rates: Low: z < −1, Mean: -1 ≤ z ≤ 1, High: z > 1.

# 1 health district and 49 municipalities are excluded from the analysis.

The table shows that only 61% of the health districts and 66% of municipalities have the same level of prevalence of the two rates. In order to have a numeric indicator of the grade of correlation, the Pearson’s coefficient of correlation between the two distributions of prevalence was calculated, and its value was 0.20 (p-value=0.0501) for the health districts and 0.24 (p-value <0.0001) for the municipalities. Although this was significant, it was very low. From the analysis of the hospitalizations it is possible to conclude that there is no significant correlation between the distribution of the prescriptions of antibacterials and the health status of the population. Since there is a relation between territory setting and prescriptions, however, it is possible to consider another element: the pediatricians.

### Analysis of the pediatricians

As anticipated, for every pediatrician of the Lombardy Region with at least 100 patients aged between 0 and 6 years, the 4 indicators A, B, C and D were calculated as described in the “Methods” section. The Pearson’s coefficient of correlation between A and B was 0.95 (p-value <0.0001) and between C and D was 0.85 (p-value <0.0001). It is therefore possible to state that the pediatricians who prescribe many antibacterials are the same pediatricians who prescribe many drugs in general. In order to classify the pediatricians the indicator B, which has a mean value of 55.4% and standard deviation of 10.3, was standardized. In all, 182 of the 1,151 (16%) pediatricians in the database are “Low prescribers”, with a rate lower than 45.1%, while 782 (68%) of the pediatricians are “Mean prescribers”, with a rate between 45.1% and 65.7%. The remaining 187 of the 1,151 (16%) pediatricians identified in our database are “High prescribers”, with a rate higher than 65.7%. The Table 3 reports the distribution by LHU.

**Table 3:** Distribution of the pediatricians by LHU and tendency to prescribe antibacterials

Prescriber*		**LHU**															**TOT**
		BG	**BS**	CO	CR	LC	LO	MN	**MI**	MI1	MI2	MB	PV	SO	VCS	VA	
**Low**	**N**	18	6	7	3	14	3	5	**64**	11	10	10	5	7	1	18	182
	**Row %**	9.9	3.3	3.8	1.6	7.7	1.6	2.7	**35.2**	6.0	5.5	5.5	2.7	3.8	0.5	9.9	100.0
**Mean**	**N**	89	78	44	28	25	21	31	93	84	67	71	49	10	6	86	782
	**Row %**	11.4	10.0	5.6	3.6	3.2	2.7	4.0	11.9	10.7	8.6	9.1	6.3	1.3	0.8	11.0	100.0
**High**	**N**	20	**43**	10	7	4	5	7	10	29	10	25	3	0	3	11	187
	**Row %**	10.7	**23.0**	5.3	3.7	2.1	2.7	3.7	5.3	15.5	5.3	13.4	1.6	0.0	1.6	5.9	100.0
**Total**	**N**	127	127	61	38	43	29	43	167	124	87	106	57	17	10	115	1,151
	**Row %**	11.0	11.0	5.3	3.3	3.7	2.5	3.7	14.5	10.8	7.6	9.2	5.0	1.5	0.9	10.0	100.0

* The 3 classification levels are based on the z-score of the prevalence rate of patients with at least one prescription of antibacterials during the year: Low: z < −1, Mean: -1 ≤ z ≤ 1, High: z > 1.

**Figure 2:**
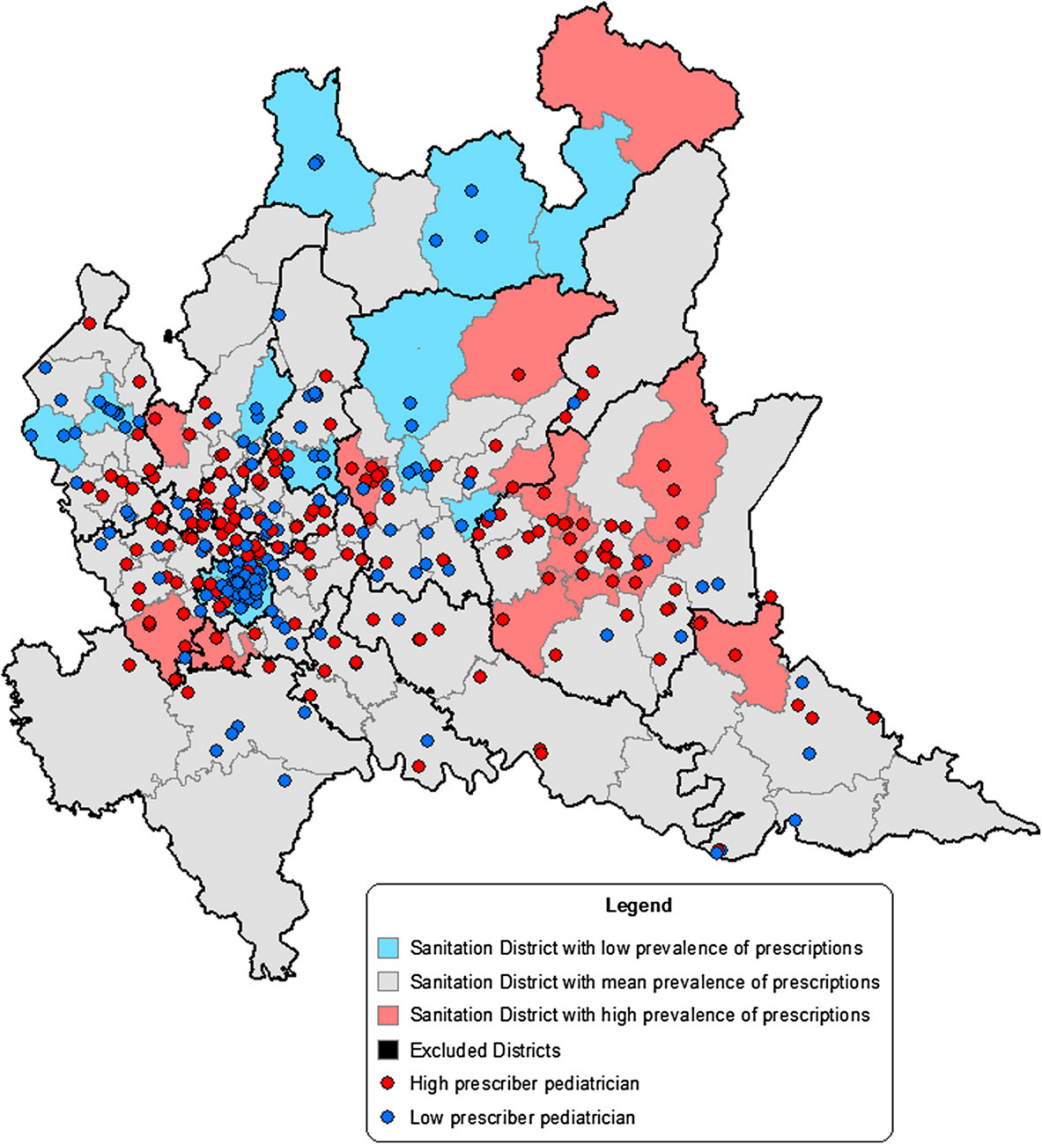
z-score of the prevalence rate of prescriptions of antibacterials and pediatricians.

The High and Low prescribers are represented on the map of the z-scores of the prevalence rate of prescriptions of antibacterials at the health district level, using the same intervals used to classify the pediatricians (z < −1, -1 ≤ z ≤ 1, z > 1). The results are shown in Figure 2.

It is possible to see that in the red health districts on the eastern side there is a concentration of “High prescribers” pediatricians, and this zone corresponds to the Brescia LHU, which has 23% of the 187 high prescribers on its territory. In the blue zone on the western side, there is a concentration of “Low prescribers”, and this zone corresponds to the Milano LHU, which has 35% of the 182 low prescribers on its territory.

### Logistic regression model

A logistic regression model was built to estimate the probability of receiving at least one prescription of antibacterials during the year (dependent variable) considering, as independent variables: age, sex, prevalence of hospitalizations in the district of residence (High, Mean, Low), prescriptive attitude of the pediatrician (High, Mean, Low), sex of pediatrician, pediatrician’s age group (three classes based on the standard deviation of the distribution), and duration of the pediatrician’s contract with the LHU (three classes based on the standard deviation of the distribution). The results are reported in Table 4.

**Table 4:** Logistic regression model

**Effect**	**Estimate**	**(95% CI)**
Age	1.13	(1.13 – 1.13)
Sex M vs F	1.12	(1.11 – 1.13)
Prevalence of hospitalizations in the district HIGH vs LOW	1.10	(1.08 – 1.13)
Prevalence of hospitalizations in the district MEAN vs LOW	1.03	(1.01 – 1.04)
**Prescriptive attitude of the pediatrician HIGH vs LOW**	**3.63**	**(3.56 – 3.70)**
**Prescriptive attitude of the pediatrician MEAN vs LOW**	**1.95**	**(1.92 – 1.98)**
Sex of the pediatrician F vs M	1.03	(1.01 – 1.04)
Pediatrician age group ADULT vs ELDERLY	1.02	(1.00 – 1.03)
Pediatrician age group YOUNG vs ELDERLY	1.07	(1.04 – 1.09)
Duration of the agreement of the pediatrician SHORT vs LONG	1.03	(1.01 – 1.05)
Duration of the agreement of the pediatrician MEAN vs LONG	1.03	(1.01 – 1.04)

The findings from the logistic regression show that the prescriptive attitude of the pediatrician is the main determinant of the antibacterial prescription. Based on this model, the probability of receiving at least one prescription of antibacterials during the year was calculated for every patient. This probability at the health districts level had a mean equal to 0.558 and standard deviation of 0.043 and follows the geographical distribution of the standardized prevalence rates at health district level shown in Figure 1A.

## Discussion

The use of thematic maps allows a more rapid and immediate reading of the results. Previous studies have analyzed the differences in drug prescription between and within Italian regions [10,17,18], but none of these compared the prescription profile at the district level or used spatial analysis. In this regard, this is the first Italian study using GIS and spatial analysis to explore the role of geographic setting in pediatric drug utilization.

On the contrary, there are several examples of drug utilization studies performing spatial analysis with the aim to examine the patterns of drug use [19-26]. Two of these are pediatric studies, one of which performed spatial cluster analyses based on simulations to identify disparities in use of psychotropic medications [23] and the other of which analyzed the regional differences in prevalence rates of antibiotics in Germany [26]. Only one study used spatial analyses to find spatial clusters with the same method employed in this study (Getis-Ord’s G statistic) [22]. All these studies highlighted the importance of the evaluation of spatial distribution in analyzing the pattern of drug consumption in a population.

Geographical variation in outpatient antibiotic prescribing was analyzed in several countries, such as Israel, Germany, Switzerland, United Kingdom, and United States [26-31]. All these studies, with the exception of the one by Koller et al. [26], concerned the overall population and mainly evaluated differences at the “macro” level (states/regions), and not at the “micro” level (municipalities). Differences in antibiotic prescriptions to children and adolescents among health districts were observed in Germany in the study by Koller et al. [26]. The prevalence of drug prescriptions ranged, in Germany, between 19.3 and 52.7%, with children living in relatively deprived areas having a 20% higher chance of receiving an antibiotic prescription. The authors from this study, however, did not consider the spatial autocorrelation or spatial clusters in the distribution of prevalence rates at the district level.

The findings from our study confirm that differences exist not only among regions and local health units, but also at the health district and municipality levels. The fact that there is spatial autocorrelation in the distribution of the prevalence of children that have received at least one prescription of antibacterials during the year 2008 emerges from this study. This distribution is not explained by the health status of the children, but it is rather the prescriptive attitude of the pediatrician (or the parents’ request to which the pediatrician agrees) that influences the distribution of the prescriptions. This fact is confirmed also by the logistic regression model that shows how the demographic variables do not significantly influence the probability of receiving at least one prescription during the year, but how it is rather the pediatricians that influence it. Previous studies performed in Italy observed differences in antibiotic prescription not correlated to differences in the hospitalization rate [10]. A possible role of physicians’ prescribing attitude in determining these differences was hypothesized [10].

Qualitative differences in the choice of antibiotic classes by pediatricians were previously observed, with physicians working in high prevalence LHUs more commonly prescribing cephalosporins than physicians working in low prevalence LHUs [9]. This latter finding reinforces the role of physicians as determinants of drug prescription.

Even if this study concerned a single Italian region, the results are relevant also beyond the Lombardy region. The importance of the local context in influencing the drug prescriptions should be investigated in all countries as part of the monitoring of drug utilization.

Findings from this study underline that priority actions for improving the rational use of antibacterials in preschool age children should concentrate on the active participation of the pediatricians in permanent education activities. These activities should be planned at the local level. Moreover, the competent authorities should increasing their efforts to limit unnecessary prescriptions and increase appropriateness of prescribing.

## Conclusions

Spatial autocorrelation in the distribution of the prevalence of children with at least one prescription of antibacterials exists, and increases with the shift from the “macro” level of the health districts to the “micro” level of the municipalities.

This study documents that the observed geographic distribution is not explained by the health status of the children, but that it is the prescriptive attitude of the pediatrician that significantly influences the geographic distribution of the rate of antibacterial prescriptions.

## Competing interests

The authors declare that they have no competing interests.

## Authors’ contributions

All the authors have made substantial contributions in the writing of the manuscript: MC analyzed the data of the study, made the maps and wrote the draft of the manuscript, RC participated in writing the manuscript and gave suggestion in the analyses, AC revisited the manuscript critically for important intellectual content from clinical point of view, AB, IF, LM provided the database, MB designed the study and revisited the manuscript critically and give the final approval of the version to be published. All authors read and approved the final manuscript.
